# Self-Organizing 3D Human Neural Tissue Derived from Induced Pluripotent Stem Cells Recapitulate Alzheimer’s Disease Phenotypes

**DOI:** 10.1371/journal.pone.0161969

**Published:** 2016-09-13

**Authors:** Waseem K. Raja, Alison E. Mungenast, Yuan-Ta Lin, Tak Ko, Fatema Abdurrob, Jinsoo Seo, Li-Huei Tsai

**Affiliations:** 1 Picower Institute for Learning and Memory, Department of Brain and Cognitive Sciences, Massachusetts Institute of Technology, Cambridge, MA, United States of America; 2 Induced Pluripotent Stem Cell Core Facility, Picower Institute for Learning and Memory, Massachusetts Institute of Technology, Cambridge, MA, United States of America; USF Health Morsani College of Medicine, UNITED STATES

## Abstract

The dismal success rate of clinical trials for Alzheimer’s disease (AD) motivates us to develop model systems of AD pathology that have higher predictive validity. The advent of induced pluripotent stem cells (iPSCs) allows us to model pathology and study disease mechanisms directly in human neural cells from healthy individual as well as AD patients. However, two-dimensional culture systems do not recapitulate the complexity of neural tissue, and phenotypes such as extracellular protein aggregation are difficult to observe. We report brain organoids that use pluripotent stem cells derived from AD patients and recapitulate AD-like pathologies such as amyloid aggregation, hyperphosphorylated tau protein, and endosome abnormalities. These pathologies are observed in an age-dependent manner in organoids derived from multiple familial AD (fAD) patients harboring amyloid precursor protein (*APP*) duplication or presenilin1 (*PSEN1*) mutation, compared to controls. The incidence of AD pathology was consistent amongst several fAD lines, which carried different mutations. Although these are complex assemblies of neural tissue, they are also highly amenable to experimental manipulation. We find that treatment of patient-derived organoids with β- and γ-secretase inhibitors significantly reduces amyloid and tau pathology. Moreover, these results show the potential of this model system to greatly increase the translatability of pre-clinical drug discovery in AD.

## Introduction

**Alzheimer’s disease (AD)** is an age-related neurodegenerative disorder associated with severe memory impairments, which has become the sixth leading cause of death in the United States (www.alz.org) [1,2]. Individuals with AD develop brain atrophy and neuronal loss, and the disease is characterized by the presence of dense extracellular deposits of amyloid plaques and neurofibrillary tangles [3–14]. Much of our understanding of the mechanisms underlying AD pathology comes from a population of individuals with early-onset familial AD (fAD). These cases harbor causal mutations involving primarily the Aβ processing enzymes, presenilin 1 and 2 (*PSEN1*, *PSEN2*), which are part of the γ-secretase complex [15–18] [15–18], or mutations within or duplications of the amyloid precursor protein (*APP*) gene itself [19–22]. Neurofibrillary tangles are comprised of intraneuronal accumulations of the microtubule-associated protein tau [19]. Tau pathology (tauopathy), consisting of aberrantly phosphorylated (pTau) and aggregated tau, is characteristic of a number of neurodegenerative disorders, including AD [23–25]. The extent of tau pathology in human AD has been shown to correlate well to cognitive impairment [26]. Many studies have shown that the aberrant phosphorylation and aggregation of tau underlie tauopathy in human brain and animal models.

Neurological conditions are difficult to study because of the limited accessibility to human brain tissue. Animal models, such as transgenic rodents, can recapitulate AD phenotypes to an extent; however, a number of drugs that have shown promising results in mouse models have, failed to prevent cognitive decline in late phase clinical trials [27–30]. There are general concerns on the validity of rodent models [31,32], and a number of genetic variants associated with increased risk for human disease are located in non-coding regions of the genome that are not well-conserved between species [33]. Thus, an alternative model for AD that exhibits pathology in human cells may better serve to predict clinical outcomes. In the last decade, the advent of induced pluripotent stem cells (iPSCs) has revolutionized human in vitro models systems [34–37]. Using this technology, we can derive specific neural cell types from patients with sporadic or familial AD, and use these cultures to both study disease mechanisms and develop novel therapies [38,39]. A number of groups have performed functional studies using iPSC-derived neural cells to model various aspects of AD pathology [40–50].

One concern with the use of in vitro systems to model AD pathology, however, is that phenotypes of aberrant extracellular protein aggregation are lost in two-dimensional (2D) cultures simply due to the lack of an interstitial compartment. To overcome this, the next logical approach is to use three-dimensional (3D) culture systems. There exist two main types of 3D culture systems: a scaffold-free 3D culture system, exemplified by self-organizing structures such as organoids or spheroids [51–53] and 3D engineering tissue, in which a variety of support materials (scaffolds or gels) provide a structure within which cells are cultivated [54–57]. One published study in this second category embedded genetically modified human neural precursor cells, which overexpressed mutant *APP* and *PSEN1*, into a Matrigel scaffold. After twelve weeks in vitro, these cultures generated AD-like phenotypes including amyloid plaque deposition and hyperphosphorylated tau [40,58]. Another group studied AD-relevant phenotypes in scaffolded 3D culture systems following exogenous amyloid β (Aβ) application [59].

While the tissue engineering approaches mentioned above do produce AD-like phenotypes and are highly amenable to experimental manipulation, the process is labor-intensive and requires the exogenous overexpression of disease-relevant mutations or factors. In the current work, we have adapted a scaffold-free culture approach to generate neural organoids from AD patient derived cells. These 3D cultures efficiently produce robust AD phenotypes, without genetic manipulation or exogenous toxins. Although neural organoids have been shown to be extremely useful for the study of neurodevelopmental phenomena and prenatal injury [51–53,60–63], no previous study had applied this system to the study of age-related neurodegeneration. We show that, by “aging” these cultures in vitro, we can observe the spontaneous emergence of hallmark AD pathologies such as amyloid aggregates and hyperphosphorylated tau. Importantly, we demonstrate that this model system is amenable to experimental manipulation, such as drug treatment, and that these phenotypes are robust enough to be recapitulated across multiple cells lines derived from different AD patients.

## Results

### Generation of organoids and analysis

To investigate the utility of such a system, we created scaffold free three-dimensional (3D) human neural organoids, from human iPSCs derived from AD patients and healthy controls and test each line for pluripotency (S1 Fig). Several protocols have been developed to create neural organoids from human pluripotent stem cells [53,64–67]. We followed the protocol published by Kadoshima *et al*. [64] with minor modifications (see Methods) and successfully created complex dense 3D neural tissues from a number of human iPSC lines from AD patients and healthy control (S2A Fig and S1 Table). We subjected the 3D cultures, heretofore referred to as organoids, to immunohistochemistry with antibodies against a neuronal protein (MAP2) as well as SOX2, a marker for neural progenitor cells, to determine the presence of neural cell types. After one month of culture, we observed the emergence of translucent regions of neuroectoderm and immunolabeling for neuronal MAP2 as well as SOX2-positive neural progenitor cells, as previously described [64] (S2 Fig). At 60 days (60d) and 90–100 days (90d) in culture, the organoids demonstrated formation of a rolling morphology structure with rosette-like neuroprogenitor rich regions in which the neuroprogenitor cells are spatially arranged in a spherical format, and unorganized regions that contain dense populations of MAP2-positive neurons (S2B Fig). With age, immunoreactivity for SOX2 appeared to decrease, while MAP2 immunoreactivity remained (S2C and S2D Fig). Since we are interested in age-related neural pathology, we found that the large regions of neuron-rich tissue produced by the modified Kadoshima et al protocol [64] best suited our modeling needs.

At 60d and 90d of culture, we subjected organoids from a healthy control (Ctrl) and a familial (fAD) patient (*APP* duplication, *APP*^Dp^1-1, reported in Israel et al., 2012) to a number of assays for AD-relevant phenotypes. We also examined additional fAD lines in (S1 Table). As the organoid continues to increase in size, the deeper regions of the tissue show evidence of necrosis, which is likely due to the absence of vasculature and lack of nutrients and oxygen penetration in the deeper layers. As the presence of necrosis could confound our measurements of AD-like phenotypes. We used immunohistochemistry for cleaved caspase 3 (CC3; S3A Fig) as an indicator of apoptotic cells and calculated the average distance between the surface and regions of increasing CC3 immunoreactivity. We found that the region of neuron-rich, CC3-sparse tissue extended an average of 250 μm from the surface into the interior of the organoid (S3A Fig). In addition, we incubated sections with secondary antibody alone (after blocking) to assess the presence of nonspecific binding. We observed that the inner tissue region also gave rise to nonspecific antibody binding compared to the more superficial region (S3B Fig). Based on these data, we established a limit of 250 μm from the surface of the organoid for our further characterization.

### Alzheimer’s disease phenotypes in organoids

#### Amyloid beta

**Fig 1:**
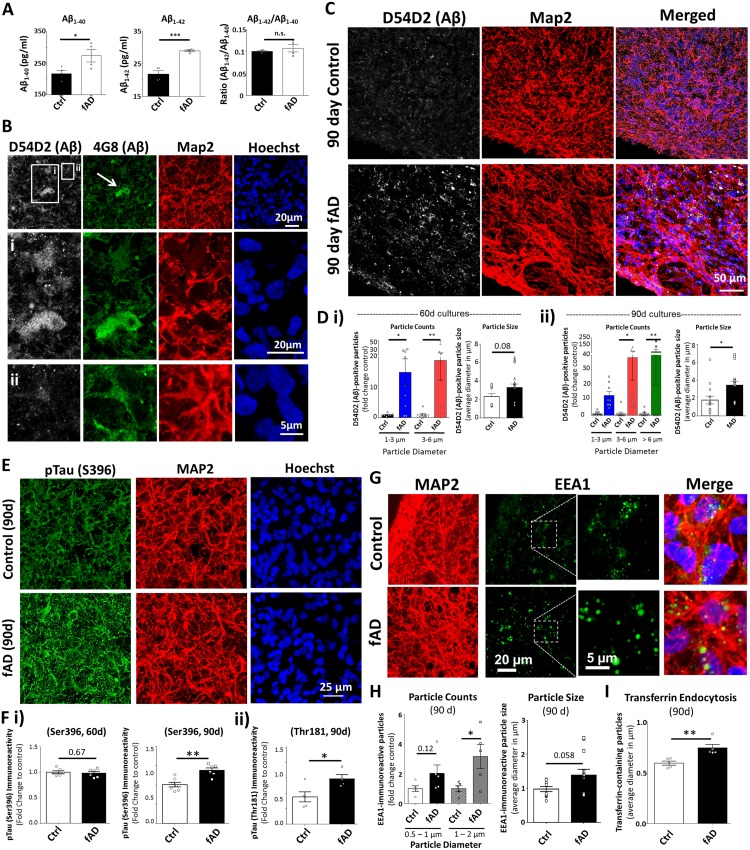
Organoids created from patient-derived iPSCs exhibit robust Alzheimer’s disease (AD)-like pathology. (A) Concentration of Aβ_1–40_ and Aβ_1–42_ from supernatant of control (Ctrl; CS-0020-01) and familial AD (fAD; *APP*^Dp^1-1) organoid cultures, measured by ELISA, as well as the ratio of Aβ_1–42_ to Aβ_1–40_ concentrations. Unpaired two-tailed t-test with equal variance: *p = 0.047 (Aβ_1–40_), unpaired two-tailed t-test with Welch’s correction for unequal variance: **p = 0.004 (Aβ_1–42_), p = 0.48 (Aβ_1-42_/Aβ_1–40_). (B) Tissue sections from fAD (*APP*^Dp^1-1) and control (Ctrl; CS-0020-01) organoids were processed for immunoreactivity against amyloid β (Aβ) using two antibodies (D54D2: white, 4G8: green), as well as antibodies against the neuronal marker MAP2 (red) and stained with the nuclear dye Hoechst (blue). Insets demonstrate Aβ immunoreactivity that appears both extracellular (i, arrow) and intracellular (ii, arrowhead) based upon MAP2 co-localization. (C) Z-projection of immunolabeled tissue sections from 90 day old Ctrl and fAD organoids showing immunoreactivity for Aβ (D45D2: white) and MAP2 (red). The edge of the tissue section is visible at the left bottom corner of each example. (D) Quantification of Aβ immunoreactivity in fAD and Ctrl organoids following 60d and 90d culture. Particle Counts: one-way ANOVA with post-hoc Fishers Least Significant Difference (LSD) test for multiple comparisons; *F* (3,28) = 4.385, ***p = 0 0.0008, R^2^ = 0.32 (i-60 days); *F* (5,43) = 3.346, *p = 0 0.012, R^2^ = 0.28 (90 days). Particle Size: Two-tailed Mann Whitney test for non-normal distributions (normality α < 0.05), **p = 0.006 (60 days), ***p = 0.001 (90 days). (E) Tissue sections from fAD (*APP*^Dp^1-1) and control (Ctrl; CS-0020-01) organoids were processed for immunoreactivity against phosphorylated Tau (pTau, green) at Serine 396 (S396) and MAP2 (red) following 90d culture. Hoechst (blue) labels cell nuclei. (F) Quantification of pTau immunoreactivity for the Ser396 at 60d and 90d, and for the Threonine 181 (Thr181) pTau at 90d. Values are plotted as mean intensity of immunoreactivity as fold change of Ctrl. Unpaired two-tailed t-test with equal variance: p = 0.67 (60 day Ser396), **p = 0.001 (90 day Ser396), *p = 0.03 (90 day Thr181). (G) Sections from fAD (*APP*^Dp^1-1) and control (Ctrl; CS-0020-01) organoids were processed for immunoreactivity against the early endosome antigen 1 (EEA1, green) and MAP2 (red). The dotted white line outlines the region of higher magnification to show EEA1 detail. (H) Quantification of EEA1 immunoreactivity in fAD and Ctrl organoids following 90d culture. EEA1 Particle Counts: one-way ANOVA with post-hoc Fisher’s LSD test for multiple comparisons; *F* (3,16) = 4.0, *p = 0.026, R^2^ = 0.43. EEA1 Particle size: unpaired two-tailed t-test with Welch’s correction: *p = 0.041. (I) Organoids from Ctrl and fAD lines were subjected to the transferrin endocytosis assay to label pools of clathrin-coated early endosomes. (Each data point represent one organoid) Quantification of the average size of transferrin-positive particles: unpaired two-tailed t-test with equal variance, **p = 0.005. Average number (count) of transferrin-positive particles, unpaired two-tailed t-test with equal variance, p = 0.64. On charts: *p < 0.05, **p < 0.01, ***p < 0.001.

Neurons derived from the *APP*^Dp^1-1 line had previously exhibited increased levels of secreted Aβ_40_ and Aβ_42_ (Israel et al., 2012). To determine whether this phenotype was perpetuated in 3D culture, we subjected culture media from fAD and control organoids to ELISA. In agreement with the previous study, we detected significantly higher levels of Aβ in the media from fAD organoids culture compared to controls (Fig 1A). We performed immunohistochemistry on fixed cryosections from control and fAD organoids to examine AD-like pathology using two different antibodies that recognize Aβ. The first antibody, 4G8 (immunoreactive against amino acid residues 17–24 of Aβ) has been widely used to label both soluble and aggregated Aβ [68]. In addition, we co-labeled sections with the anti-Aβ antibody D54D2, which recognizes several isoforms of amyloid (Aβ_37_, Aβ_38_, Aβ_39_, Aβ_40_, and Aβ_42_). In 90d organoids, we detected aggregates that were immunopositive for both Aβ antibodies (Fig 1B), while 4G8 immunoreactivity appeared against intracellular amyloid that co-localizes with MAP2 as well as putative extracellular aggregates (Fig 1B, S4A Fig and S1 and S2 Movies). We then used the D54D2 antibody to quantify the size and number of Aβ aggregates. The 90d fAD organoids contained numerous Aβ aggregates compared to the control organoid tissue (Fig 1C). Analysis at 60d and 90d of culture identified a progressive increase in number and size of Aβ aggregates in the fAD organoids compared to controls (Fig 1D and S4B Fig). Additionally, we performed Western blot on the 90d whole organoids lysates and observed increased levels of Aβ oligmers in fAD organoids compared to controls (data not shown). These data demonstrate the presence of robust and spontaneous Aβ aggregation in AD patient-derived organoid culture models that appears to develop in a time-dependent manner.

#### Hyperphosphorylated Tau (pTau)

Another hallmark of AD is the abnormal phosphorylation, mislocalization, and aggregation of the tau protein. Beta-plated sheets of hyperphosphorylated tau (pTau) leads to the disruption of neuronal microtubule assemblies and neurodegeneration [19]. To examine control and fAD organoids for the presence of tau pathology (tauopathy), we conducted immunohistochemistry in organoid sections using antibodies against pTau (Ser396 or Thr181). At 90d, the fAD organoids exhibit significantly greater pTau immunoreactivity than did control sections (Fig 1E and 1F and S4C Fig). Interestingly, there was no significant difference in pTau levels between fAD and control organoids at 60 days (Fig 1F). This is in contrast to the amyloid phenotype, in which more Aβ aggregates were observed at both 60d and 90d in the fAD organoids (Fig 1D). The Thioflavin-S dye binds aggregates of β-pleated sheets and is used as an indicator of tau pathology in human brain and mouse models [69]. We observed a greater total area covered by Thioflavin-S dye labeling in fAD organoids compared to controls, as well as a higher number of Thioflavin-S positive particles (S4D and S4E Fig). Together, these various measures demonstrate that the AD patient-derived organoids have the power to recapitulate both amyloid and pTau phenotypes.

#### Endosome abnormalities

Endosome abnormalities are another common cellular phenotype in AD. Enlarged RAB5-positive early endosomes have been observed in mouse models and in the brains of sAD and fAD patients [70,71]. Such enlarged endosomes may contain aberrantly phosphorylated APP and aggregated Aβ [72]. iPSC-derived neurons created from sAD, fAD, and frontotemporal dementia (FTD) patients also show increases in the number of medium to large RAB5-positive endosomes [44] and defects in endosome trafficking [43,48]. To examine endosome phenotypes in iPSC-derived organoids, we labeled control and fAD organoid sections with antibodies against the early endosome marker 1 (EEA1; Fig 1G). The fAD organoids trended towards an increase in the number of small endosomes (<1 μm in diameter) and exhibited a significantly higher number of large (1–2 μm) endosomes compared to controls. Additionally, we observed a trend towards an increased overall size for EEA1-positive endosome particles in the fAD organoids (Fig 1H). To functionally assess endosome trafficking in the control and fAD organoids, we employed the transferrin endocytosis assay, in which labeled transferrin is taken up by live cells via clathrin-mediated endocytosis [73]. Following incubation with Alexa 488-conjugated transferrin, we observed a significant increase in the size of transferrin-containing endosomes in the fAD line, compared to controls (Fig 1I). Together, these results demonstrate that AD patient-derived organoids display a third hallmark AD: abnormal endosome morphology and recycling.

### Organoids from multiple AD lines recapitulate AD pathology

**Fig 2:**
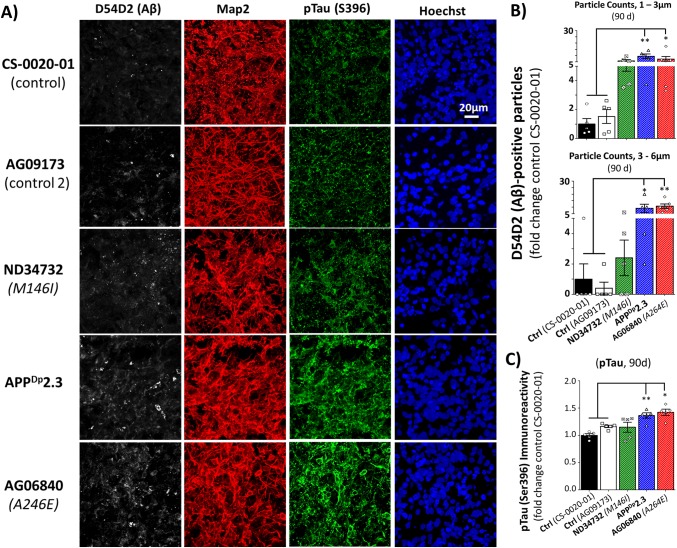
Organoids created from different lines of AD patient iPSCs exhibit AD phenotypes. (A) Tissue sections from fAD (*APP*^Dp^2-3, ND34732, AG068840) and control (Ctrl; CS-0020-01, AG09173) organoids were processed for immunoreactivity against Aβ (D45D2, white), MAP2 (red), and pTau (S396, green) and labeled with the nuclear dye Hoechst. (B) Quantification of Aβ immunoreactivity in fAD and Ctrl organoids following 90 days of culture. Values between the two control lines did not significantly differ. Number of Aβ-positive aggregates in two size classes (Particle Counts): one-way ANOVA with post-hoc Tukey’s multiple comparisons test; *F* (4,21) = 6.15, **p = 0.0019, R^2^ = 0.5396 (1–3μm); *F* (4,21) = 7.95, ***p = 0.0005, R^2^ = 0.6024 (3–6 μm). (C) Quantification of the average intensity of pTau Ser396 immunoreactivity as a fold change of Ctrl in fAD and Ctrl organoids following 90 days of culture. Values between the two control lines did not significantly differ. (Each data point represent one organoid). One-way ANOVA with post-hoc Tukey’s multiple comparisons test; *F* (4,20) = 9.629, ***p = 0.0002, R^2^ = 0.6582. On charts: *p < 0.05, **p < 0.01, ***p < 0.001.

To ascertain whether the AD phenotypes of Aβ aggregation and Tau hyperphosphorylation are generalizable to fAD organoids from different sources, rather than being a phenomenon of the *APP*^Dp^1-1 line, we examined additional organoids created from patient-derived and healthy control iPSCs (S1 Table). At 90d of culture, we processed and analyzed organoids created from an additional *APP* duplication line (*APP*^Dp^2-3; [44]) two *PSEN1* fAD mutant lines (*PSEN1*^M146I^, *PSEN1*^A264E^; Fig 2A, S1 Table). Organoids derived from fAD patients with *APP* duplication (*APP*^Dp^2-3) or the *PSEN1*^A264E^ mutation exhibited increased numbers of Aβ aggregates verses the control lines (Fig 2B). While the fAD organoids from *PSEN1*^M146I^ patient cells exhibited a trend towards higher amyloid levels, this was not significant. We also examined organoids from these additional fAD lines for the presence of pTau immunoreactivity. Similar to the pattern observed with amyloid aggregation, the *APP*^Dp^2-3 and the *PSEN1*^A264E^ fAD organoids exhibited increased pTau (Ser396) immunoreactivity at 90 days of culture, while the *PSEN1*^M146I^ organoids did not differ from control (Fig 2C).

### Attenuation of AD pathology in neural organoids by β- and γ-secretase inhibitor treatment

**Fig 3:**
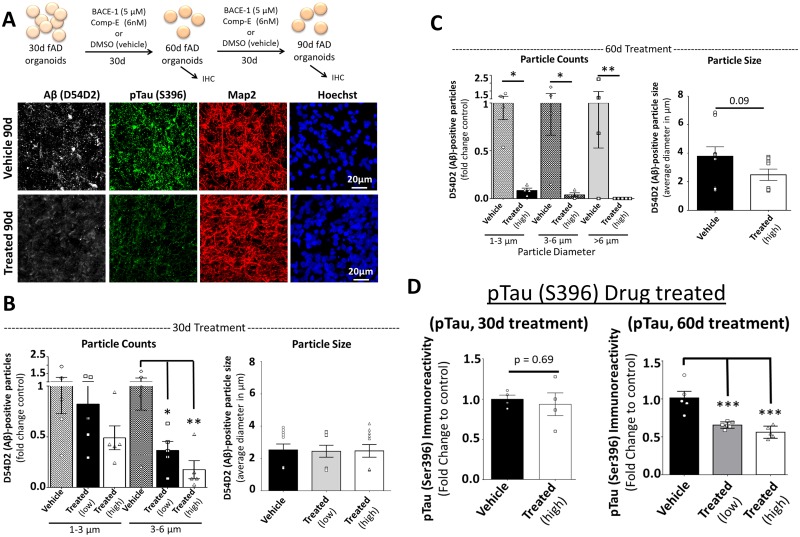
Organoids created from AD patient iPSCs respond to compound treatment. (A) Schematic of beta (BACE-1) and gamma (Comp-E) secretase inhibitor treatment (top). At 30 days of culture, fAD (*APP*^Dp^1-1) organoids were treated with low dose (BACE-1, 1μM and Comp-E, 3nM) or high dose (BACE-1, 5 μM and Comp-E 6nM) combined compounds, or equivalent DMSO vehicle. Following 30 or 60 days of culture and drug treatment, organoids at 60 and 90 days of culture, respectively, were processed for immunohistochemistry (IHC). Tissue sections from fAD (*APP*^Dp^1-1) and control (Ctrl; CS-0020-01) organoids were processed for immunoreactivity against Aβ (D45D2, white), pTau (Ser396, green), and MAP2 (red). Examine images are from 90 day organoids. (B) Quantification of Aβ particle number and size in compound treated and fAD organoids following 30 days of administration. Number of Aβ-positive aggregates in two size classes (Particle Counts): one-way ANOVA with Fishers LSD test for multiple comparisons; *F* (5,24) = 3.58, *p = 0.014, R^2^ = 0.4296. Particle size: one-way ANOVA with Kruskal-Wallis test for non-normal distribution (α < 0.05), p = 0.475. (C) Quantification of Aβ particle number and size in treated (high dose) and untreated fAD organoids following 60 days of compound administration. Number of Aβ-positive aggregates in three size classes (Particle Counts): one-way ANOVA with Fishers LSD test for multiple comparisons; *F* (5,19) = 5.02, **p = 0.004, R^2^ = 0.5691. Particle size: Mann-Whitney two-tailed test for non-normal distribution (α < 0.05), p = 0.09. (D) Quantification of the average intensity of pTau Ser396 immunoreactivity as a fold change of Ctrl in fAD organoids following 30 and 60 days of compound treatment. 30 day treatment. (Each data point represent one organoid). Unpaired two-tailed t-test with equal variance, p = 0.69. 60 day treatment: one-way ANOVA with Tukey’s multiple comparisons test, F (2,13) = 19.82, ***p = 0.0001, R^2^ = 0.7530. On charts: *p < 0.05, **p < 0.01, ***p < 0.001.

These data indicate that organoids created from multiple fAD patient iPSC lines demonstrate robust and relevant AD-like phenotypes. To determine whether these phenotypes are indeed a result of altered Aβ production, we treated the fAD organoids with two compounds well known to reduce amyloid aggregation: the γ-secretase inhibitor Compound E (Comp-E; γ2) and a BACE-1 β-secretase inhibitor (β-Secretase Inhibitor IV, EMD Millipore) [40,74]. These experiments also allow us to determine the feasibility of pharmacologic manipulation in the organoid cultures. Comp-E and β-Secretase Inhibitor IV, or DMSO vehicle, were added to the culture media of 30-day-old fAD organoids and replenished with every media change (Fig 3A). At day 60 (30 days of treatment) we assessed Aβ and pTau pathology (Fig 3A) and showed that compound treatment significantly reduced the number of amyloid aggregates in the fAD organoids in dose dependent manner, compared to vehicle treated fAD tissue (Fig 3B). This reduction was particularly evident in the in 90-day-old fAD organoids treated for 60-days (Fig 3C). In contrast to the dramatic reduction of Aβ aggregation with compound treatment, immunoreactivity for pTau was unaffected following 30 days of treatment compared to vehicle-treated controls (Fig 3D). However, following 60 days of treatment, the 90 day-old organoids showed significantly less pTau immunoreactivity compared to vehicle-treated controls in dose dependent manner (Fig 3D). These results indicate that specific AD-like phenotypes observed in patient iPSC-derived organoids can be ameliorated with drug treatment, suggesting that this system is amenable to compound testing.

## Discussion

In diseases such as AD that are characterized by protein aggregation, the presence of a true interstitial compartment is important for modeling pathology. Previous three-dimensional (3D) tissue engineering approaches have embedded neural progenitors or cell types of interest in a matrix or a scaffold [40,58]. While these ingenious approaches can model AD phenotypes, they do not recapitulate spontaneous pathology resulting from endogenous cellular characteristics, but rather necessitate the overexpression of fAD genes. In the current work, we took advantage of scaffold-free 3D tissue culture protocols to create neural organoids using iPSCs from fAD patients and healthy controls. The dense nature of these cultures likely facilitates protein aggregation, while remaining amenable to experimental manipulation such as compound treatment.

The ease with which large numbers of these organoids can be created, and their ability to respond to compound treatment, open the possibility for phenotypic and mechanistic compound screening in complex human tissue models of multiple neurodegenerative diseases. With iPSC technology, we can access patient-derived cells carrying AD-associated variants in a number of genes, which are difficult to model as many are non-coding and/or associated with more than one significant single-nucleotide polymorphism (SNP) [75]. Moreover, rapid improvements in gene editing technology allows researchers to create isogenic iPSC and ESC lines that carry disease-associated coding variants, both well-known (such as in the *APOE* gene, [76,77] or emerging (i.e. *SORL1* [50,78], *TREM2*, [79]). Together, these advancements allow us to conceive of systems in which we can test candidate therapies on complex neural tissue systems targeted to defined subpopulations of late-onset or sporadic AD patients.

One powerful aspect of the current model is the spontaneous appearance of both [1] amyloid and tau pathology, and the distinct timeline on which these phenotypes appear. Modeling both these facets of AD has been challenging in mouse models. Most mouse AD models must carry multiple transgenes to achieve robust amyloid phenotypes, and rarely have significant tau pathology or neuronal loss (for review see [31]). Mouse models of tauopathy overexpress mutated human *MAPT* (which is causal for frontotemporal dementia, not AD) to induce tau pathology [80,81], and do not exhibit amyloid aggregation. Using the organoid model, we observed that amyloid pathology emerges prior to significant tau hyperphosphorylation in neural tissue derived from fAD patients carrying a duplication of the *APP* gene. While the sequential emergence of amyloid and tau pathology in human AD remains somewhat controversial [82–85], this timing is in close agreement with that observed in the scaffolded 3D cultures of Choi *et al*. 2014; [40]. Additionally, the inhibition of Aβ production using β and γ-secretase inhibitors reduced tau hyperphosphorylation only at the later time point of treatment, after Aβ reduction was observed. Thus, AD-relevant phenotypes of Aβ accumulation emerge prior to tauopathy in this model. Moreover, the reductions in Aβ accumulation that occur from the inhibition of APP processing lead to a dose- and time-dependent amelioration of tauopathy in the fAD organoids, suggesting a causal relationship between these relevant pathologies in the neural organoid model.

In the current work, we focused our efforts on determining whether or not these organoid systems could model age-related AD-like pathology. The pioneering works that we drew our techniques from used the organoid system as a means to study neurodevelopment [53,64,66,67]. Since work by several groups has suggested that iPSC reprogramming “re-sets” the epigenome, and that other phenotypes associated with cellular aging, such as mitochondrial function and telomere length, are returned to a “juvenile-like” state [86,87], the obvious question is: to what extent can we model phenotypes associated with aging in human neural cells? While we observe robust AD-like phenotypes that increase with “age” in the organoids, the extent to which the organoid tissue represents the aged human brain has not been examined. We believe that the scaffold-free three dimensional model has good potential for studying neurological diseases. This will be important for future works to use this model system to examine other AD related phenotypes, such as neuroinflammation, gliosis, DNA damage, U1 tangles [88] and synaptic dysfunction. Also, without a means of tissue perfusion, the organoid suffers from the same issues as primary slice culture, in that the distance from the culture medium interface is correlated with tissue necrosis. There is currently great interest in the combination of three-dimensional neural culture systems with artificial blood-brain barrier technology [89–91], to address this issue.

## Experimental Procedures

### Maintenance of PSC and 3D culture differentiation

Induced pluripotent stem cells (iPSCs) were created from human fibroblasts (S1 Table). Two of the iPSC lines carrying duplications in the gene for Amyloid Precursor Protein (*APP*; *APP*^Dp^1-1 and *APP*^Dp^2-3) were provided by Dr. Lawrence Goldstein at the University of California, San Diego, and have been described previously [44]. One control iPSC line (AG09173) was kindly provided by Dr. Bruce A. Yankner at McLean Hospital and Harvard Medical School. The other lines were generated from fibroblasts at the Picower institute of Learning and Memory, iPSC core facility at the Massachusetts Institute of Technology (MIT) using Sendai virus to overexpress OCT4, SOX2, KLF4, and c-MYC. S1 Table. details the sources and attributes of the cells used in this study. Pluripotency was confirmed by immunocytochemistry for TRA-1-81 and TRA-1-60 (S1 Fig). All reagents were purchased from Life Technologies Corporation, Grand Island, NY, unless mentioned otherwise. iPSCs were cultured on irradiated mouse embryonic fibroblasts (MEFs, MTI-GlobalStem, Gaithersburg, MD) in DMEM/F12 media supplemented with knockout serum replacement (KSR, 20% v/v), non-essential amino acids (NEAA-1X), GlutaMAX (1X), beta-Fibroblast Growth Factor (FGF2, PeproTech, Inc, Rocky Hill, NJ) and 2-mercaptoethanol (0.1 mM). The quality of cells was monitored daily and differentiated cells were mechanically removed under a light microscope in a biosafety hood. iPSCs were culture up to 80% confluence and dissociated into single cell suspension after treated with Accutase (diluted in PBS (1.5:1) containing Rock inhibitor (Y-27632 dihydrochloride, Tocris Biosciences, Minneapolis, MN) to improve cell survival. The MEFs and iPSCs were separated by plating the single cell suspension onto 0.1% gelatin (0.1%, EMD Millipore, Billerica, MA) coated dishes for 45 min, after which time the MEFs attach to the substrate and the non-adherent iPSCs were collected.

To create 3D cultures, or neural organoids, we followed a published protocol [64] with some modifications. Embryoid bodies (EBs) were formed by loading 12,000 iPSCs per well into 96-well plates with cone-shaped wells (Nunc^®^ 96-well Conical Bottom plates, VWR International, Bridgeport, NJ) pre-coated with Pluronic acid (F-127, 1%, Sigma-Aldrich, Natick, MA). The 96 well plates were transferred to an incubator at 37 C° with 95% relative humidity and 5% CO_2_. Media used in EB culture consisted of Glasgow-MEM supplement with KSR (20% v/v), Sodium Pyruvate (1X), NEAA (1x), 2-mercaptoethanol (0.1mM), Rock inhibitor (20 μM), TGFβ-inhibitor (SB431532 compound, Tocris Biosciences, Minneapolis, MN; 5 μM), Wnt-inhibitor (IWRe1 compound, Tocris Biosciences, Minneapolis, MN; 3 μM). The EBs maintained in this medium for 18–20 days, with Dorsomorphin (BMP signal inhibitor, Tocris Biosciences, Minneapolis, MN; 2 μM) added to the culture for the first three days to promote a neuronal lineage. Media was replaced every other day. The edges of the EBs began to appear translucent around day 10, and the tissue grew to be large than 0.6 mm in diameter by day 18. The cell aggregates, or organoids, were transferred to non-adherent petri-dishes (EZsphere dish) to prevent fusion of separate organoids, and cultured in a medium designed to promote neuroepithelial formation, which consisted of DMEM/F12 supplemented with Chemically Defined Lipid Concentrate (1X) and N2-supplement (1X) and maintained in an incubator with 5% CO_2_ and 40% Oxygen. The aggregates were kept in this media for 15 to 20 days, at which time heparin (5 μM, Sigma-Aldrich, Natick, MA), FBS (10% v/v, Gemini Bio-Products, West Sacramento CA), and Matrigel (final 1% v/v, Corning Incorporated—Life Sciences, Oneonta, NY) were added to the medium. On day 70 the amount of Matrigel was increased to 2% and B27 supplement (1X) was added to the medium. The aggregates were maintained in this final medium for the remained of the culture period. Medium in the culture dishes was replaced every 4 to 5 days.

### Tissue processing and Immunohistochemistry

At pre-defined time points (60 days (60d) and 90–100 days (90d)) the organoids were fixed overnight in 4% paraformaldehyde (diluted from 32%, Electron Microscopy Sciences, Hatfield, PA) and cryoprotected in a 30% sucrose solution prior to embedding in optimal cutting temperature compound (OCT), VWR International, Bridgeport, NJ). Frozen tissue was sectioned at 30 μm using a cryostat and collected on ultra-frosted glass microscope slides. Sections were stored at -20C°. For immunolabeling, sections were permeabilized for 30 minutes in phosphate-buffered saline (PBS) containing 0.3% Triton-X100 (Sigma-Aldrich, Natick, MA) and then blocked in 10% v/v horse serum solution in PBS contain 0.1% Triton-X100 (PBST) for 1 hour followed by incubation with primary antibodies overnight at 4°C in PBST containing 5% horse serum (source). Sections were received three 15 min washes in PBST containing 5% horse serum. Secondary antibodies was prepared in PBST containing 5% horse serum and Hoechst (for nuclear immunoreactivity, source) and sections were incubated in secondary for 1 hour at room temperature before washing as before. Sections were then coverslipped in Fluoromount-G (Electron Microscopy Sciences, Hatfield, PA) mounting medium and the edges sealed with nail polish. For consistency, sections from control (Ctrl) and familial Alzheimer’s disease (fAD) organoids were processed in the same batch. The primary antibodies used with following dilutions: Microtubule-associated protein 2, MAP2 (chicken/1:400, Biolegend, San Diego, CA), 4G8 (mouse/1:400, Biolegend, San Diego, CA), β-amyloid (D54D2) (rabbit/1:400, Cell Signaling Technology, Danvers, MA), phosphorylated Tau (Ser396 –PHF13 and Thr181—D9F4G) (mouse and rabbit/1:400, Cell Signaling Technology, Danvers, MA), Early endosome antigen 1 (EEA1) (mouse/1:500, BD Biosciences, San Jose, CA) and Cleaved Caspase-3 (Asp175) (rabbit, 1:500, Cell Signaling Technology, Danvers, MA). Secondary anti-mouse, anti-rabbit, and anti-chicken antibodies conjugated to cyanine dyes (Cy2, Cy3 and Cy5, respectively) were purchased from Jackson Immuno Research laboratories (West Grove, PA). Twenty-four hours after coverslipping, sections were imaged using a laser scan confocal microscope (LSM710, Carl Zeiss) by researchers blind to experimental condition. For each tissue section and organoid, we imaged five different regions. Each treatment and conditioned contained between five and eight organoids.

### Thioflavin-S staining

All reagents for Thioflavin-S staining were purchased from Sigma-Aldrich, Natick, MA, unless otherwise indicated. Sections were rinsed with PBS for 5 minutes and then incubated in 0.05% potassium permanganate solution for 20 minutes followed by two washes in PBS and de-staining in 0.2% potassium metabisulfite and 0.2% oxalic acid (until the brownish color from the potassium permanganate is removed, less than 1 minute). The potassium metabisulfite/oxalic acid was washed out using PBS and sections were incubated in freshly-prepared 0.02% Thioflavin-S solution (in 40% ethanol) for four minutes in the dark. The remaining steps took place in the dark. Following staining, sections were developed with 50% ethanol for 15 minutes and followed by three time PBS wash and one time deionized water wash. Slides were coverslipped in Fluoromount-G and edges were sealed with nail polish. Fluorescent Thioflavin-S signals were imaged using a laser scan confocal microscope (LSM710, Carl Zeiss) by researchers blind to experimental condition. For each tissue section, we imaged five different regions.

### Image processing

Images were processed using ImageJ software (NIH) by researchers blind to experimental condition. Measurements consisted of particle counts and size for β-amyloid (Aβ), EEA1, and transferrin immunoreactivity, as well as signal intensity for pTau. Particle count and size were measured using a macro that converted the desired image channel into grayscale with automatic thresholding, inverting, and calling up the Analyze Particle measurement tool. Particle counts were binned by size range. Images were decoded and plotted as fold increase compared to control. For pTau immunoreactivity, the mean intensity of the entire image was measured and plotted as fold increase compared to control.

### Enzyme-linked immunoabsorbent assay (ELISA)

Aβ concentration was measured from organoid supernatants using commercially available ELISA kit for Aβ (1–40) and Aβ (1–42) (Life Technologies Corporation, Grand Island, NY) following the manufacturer’s protocol. Briefly, media samples were incubated (4 hours) in primary antibodies against the COOH-terminus of the 1–40 or 1–42 Aβ sequence in pre-coated 96 well plates (pre-coated with monoclonal antibody specific to human Aβ 1–40 or 1–42) followed by aspiration and four washes (in washing buffer) prior to incubation with HRP-conjugated secondary antibodies followed by aspiration, four washes, and addition of HRP substrate (3,3',5,5'-tetramethylbenzidine). The reaction was stopped using 1 N sulfuric acid and absorption was measured at 450 nm in an EnSpire plate readers (Perkin Elmer). Absolute values were calculated from a standard curve and plotted as either picogram/ml (pg/ml) or Aβ42/40 ratio per organoid.

### Transferrin assay

Live organoids were cut in small pieces using surgical blade and cultured in a solution of transferrin conjugated to Alexa Fluor-488 (Alexa Fluor-488, Life Technologies Corporation, Grand Island, NY). Following a 10 minute incubation in 200 μg/ml transferrin solution in media, organoids were washed three time in PBS and fixed using 4% paraformaldehyde. The fixed organoids were then washed with PBS, stained with Hoechst, and whole-mounted onto glass coverslip. The outer (flat) surface of tissue sitting on the glass coverslip were imaged using confocal microscope. The images were processed in ImageJ, by removing the background (thresholding) and counting the size of Alexa Fluor-488 positive particles.

### Drug treatment

Organoids were treated for 30 or 60 days with a combination of beta secretase (BACE-1) inhibitor (β-Secretase Inhibitor IV, EMD Millipore) and gamma secretases inhibitor (Compound E; EMD Millipore) or equivalent DMSO vehicle. Each compound was diluted into culture medium from a 5 mM DMSO stock that had been stored at -20°C. The concentrations of BACE-1 and Comp-E that were used were 5 μM and 6 nM or 1 μM and 3 nM, respectively. Vehicle-treated cultures received the same concentration of DMSO. Drug treatment was begun at day 30 of the culture and maintained until the endpoint at 60 or 90 days of culture. Media containing treatment or vehicle was replaced every 4 to 5 days. Organoids were processed for immunohistochemistry as described above.

### Statistical Analysis

The data were plotted using GraphPad Prism software. Groups were compared via Student’s two tailed t-test (two groups) or one-way analysis of variance (ANOVA; multiple groups). Please see figure legends for details.

## Supporting Information

S1 Fig Immunoreactivity for markers of pluripotency. iPSC colonies were plated onto MEF-covered glass coverslips in hES maintenance media and subjected to live immunocytochemistry for the pluripotency markers Tra-1-60 and Tra-1-81 three days after plating. Cells were then fixed and stained with the nuclear dye Hoechst (blue) and imaged using a confocal microscope. All lines tested were immunopositive for Tra-1-60 (left, green) and Tra-1-81 (right, green).
(TIF)

S2 Fig Work flow for three-dimensional (3D) neural organoid culture and immunohistochemistry with cell type-specific markers. (A) The process of 3D culture protocol begins with iPSCs that are dissociated into a single-cell suspension and induced to form embryoid bodies (EBs) in a 96 well format (one EB per well). EBs were then differentiated over a period of weeks to months. (B) Images from organoids cultured for fewer than 60 days: phase contrast images (left) and sections immunostained using antibodies against markers of neural progenitor cells (SOX2, white) and neurons (MAP2, red), as well as the nuclear stain Hoechst (blue). In the images, one can see the (i) rolling morphology structure with organized translucent layers previously reported by the Sasai group [64], (ii) regions of the organoid with a rosette-like structure dense in neural progenitors, and (iii) unorganized translucent regions. (C and D) At 60 and 90 days in culture, organoids exhibit SOX2 (white) and MAP2 (red) immunoreactivity that is variable by region.
(TIF)

S3 Fig Immunoreactivity for cleaved-caspase 3 (CC3) and non-specific antibody binding define deep regions of necrotic tissue in the organoid. (A) A quantitative analysis of CC3 immunoreactivity leads us to limit our analyses to the peripheral 250 μm of MAP2-rich (red) and CC3-poor (white) region of the organoid. CC3- and Hoechst (nuclei)-positive cells were counted in bins of 30μm from the surface of the organoid in three replicates. (B) Organoid sections were blocked and incubated with secondary antibodies alone to determine the degree of non-specific labeling (left column = 90d whole organoid images). Regions of interest within the first 250 μm (right column) and deep (middle column) in the organoid tissue were analyzed for signal from secondary antibodies (anti-mouse-Cy2, green; anti-rabbit-Cy3, red; anti-chicken, white).
(TIF)

S4 Fig Immunohistochemistry for amyloid beta and pTau at Threonine 181 (Thr181) and quantification for Thioflavin-S labeling in Ctrl and fAD organoids. (A) Additional images from tissue sections from fAD (*APP*^Dp^1-1) and control (Ctrl; CS-0020-01) organoids processed for immunoreactivity against amyloid β (Aβ) using two antibodies (D54D2: white, 4G8: green), as well as antibodies against the neuronal marker MAP2 (red) and stained with the nuclear dye Hoechst (blue). (B) Table of average particle number observed per 250 x 250 μm^2^ area in control and fAD organoids. (C) Tissue sections from fAD (*APP*^Dp^1-1) and control (Ctrl; CS-0020-01) organoids were processed for immunoreactivity against phosphorylated Tau (pTau, green) at Thr181 and MAP2 (red) following 90 days of culture. Hoechst (blue) labels cell nuclei. (D) Representative images of fAD (*APP*^Dp^1-1) and control (Ctrl; CS-0020-01) organoids section stained with Thioflavin-s dye for beta-pated sheet structure. (E) Quantification of fluorescent Thioflavin-S in tissue sections from fAD (*APP*^Dp^1-1) and control (Ctrl; CS-0020-01) organoids after 90 days of culture (Each data point represent one organoid). Measurements consist of the percent of the visual field covered by Thioflavin-S signal (left) and the number of distinct Thioflavin S-positive particles (right). Unpaired two-tail t-test with equal variance, **p = 0.0074 (% Area Covered), *p = 0.022 (Particle Count). On charts: *p < 0.05, **p < 0.01.
(TIF)

S1 Movie Extracellular amyloid aggregates. 30 μm thick (90d) fAD organoids (made from App^Dp^1-1) were stained for amyloid beta using two different antibodies; 4G8 (green) and D54D2 (White) along with neuronal marker MAP2 and Hoechst for nuclear staining. The images were converted into movie (2 frames per second) using imageJ (NIH).
(AVI)

S2 Movie Extracellular amyloid aggregates. 90d fAD organoids (made from App^Dp^1-1) were immunolabeled using antibodies against amyloid beta (D54D2, White) along with neuronal marker MAP2 and Hoechst for nuclear staining. The images were converted into movie (6 frames per second) using imageJ (NIH).
(AVI)

S1 Table Cell lines used in the current study. (PDF)
