# Supplementary figures and images for: Self-Organizing 3D Human Neural Tissue Derived from Induced Pluripotent Stem Cells Recapitulate Alzheimer’s Disease Phenotypes
Source: PLoS One. 2016 Sep 13;11(9):e0161969. doi: 10.1371/journal.pone.0161969 (PMC5021368; doi:10.1371/journal.pone.0161969)

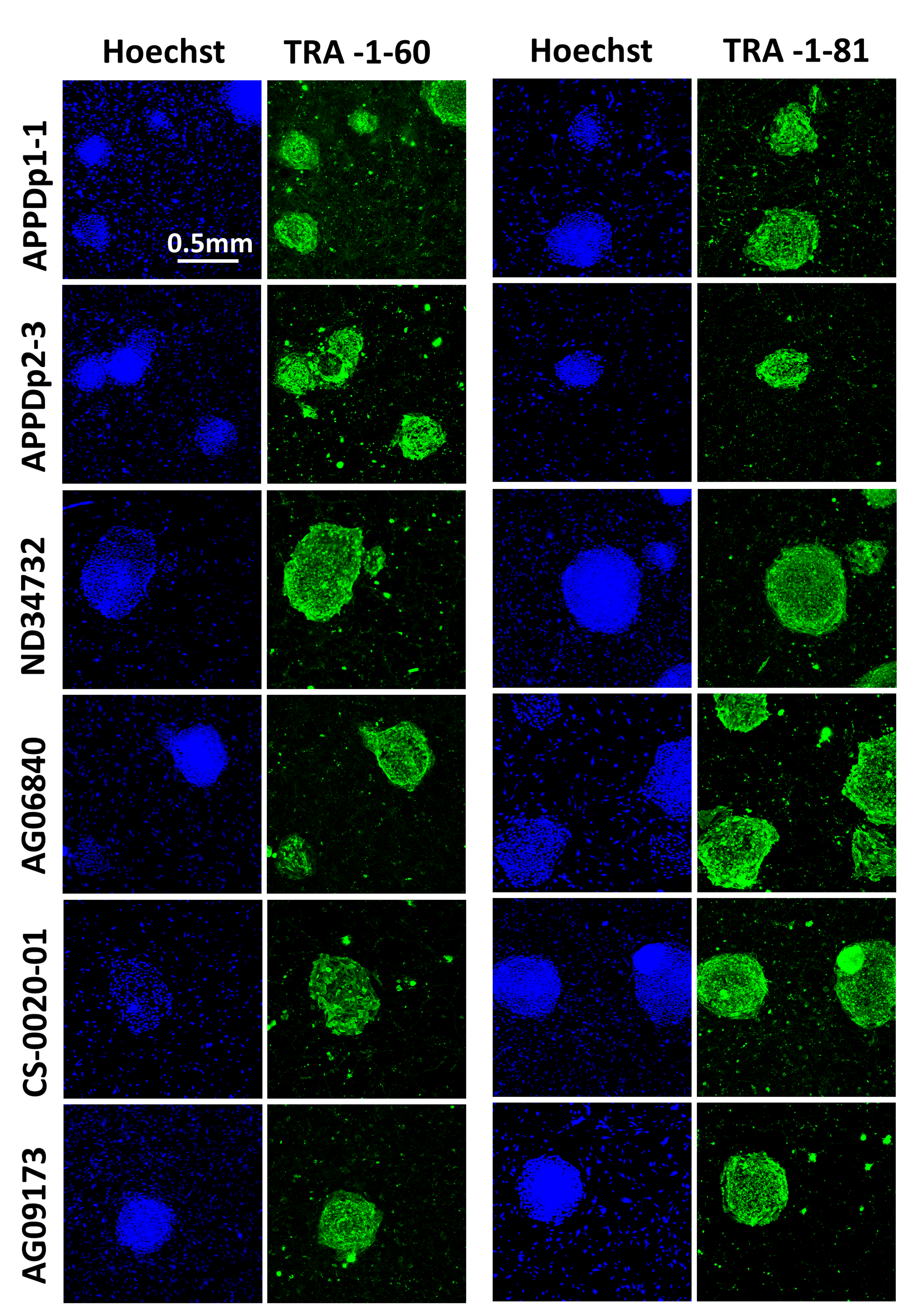

Supplement: S1 Fig — iPSC colonies were plated onto MEF-covered glass coverslips in hES maintenance media and subjected to live immunocytochemistry for the pluripotency markers Tra-1-60 and Tra-1-81 three days after plating. Cells were then fixed and stained with the nuclear dye Hoechst (blue) and imaged using a confocal microscope. All lines tested were immunopositive for Tra-1-60 (left, green) and Tra-1-81 (right, green). (TIF) [file pone.0161969.s001.tif]

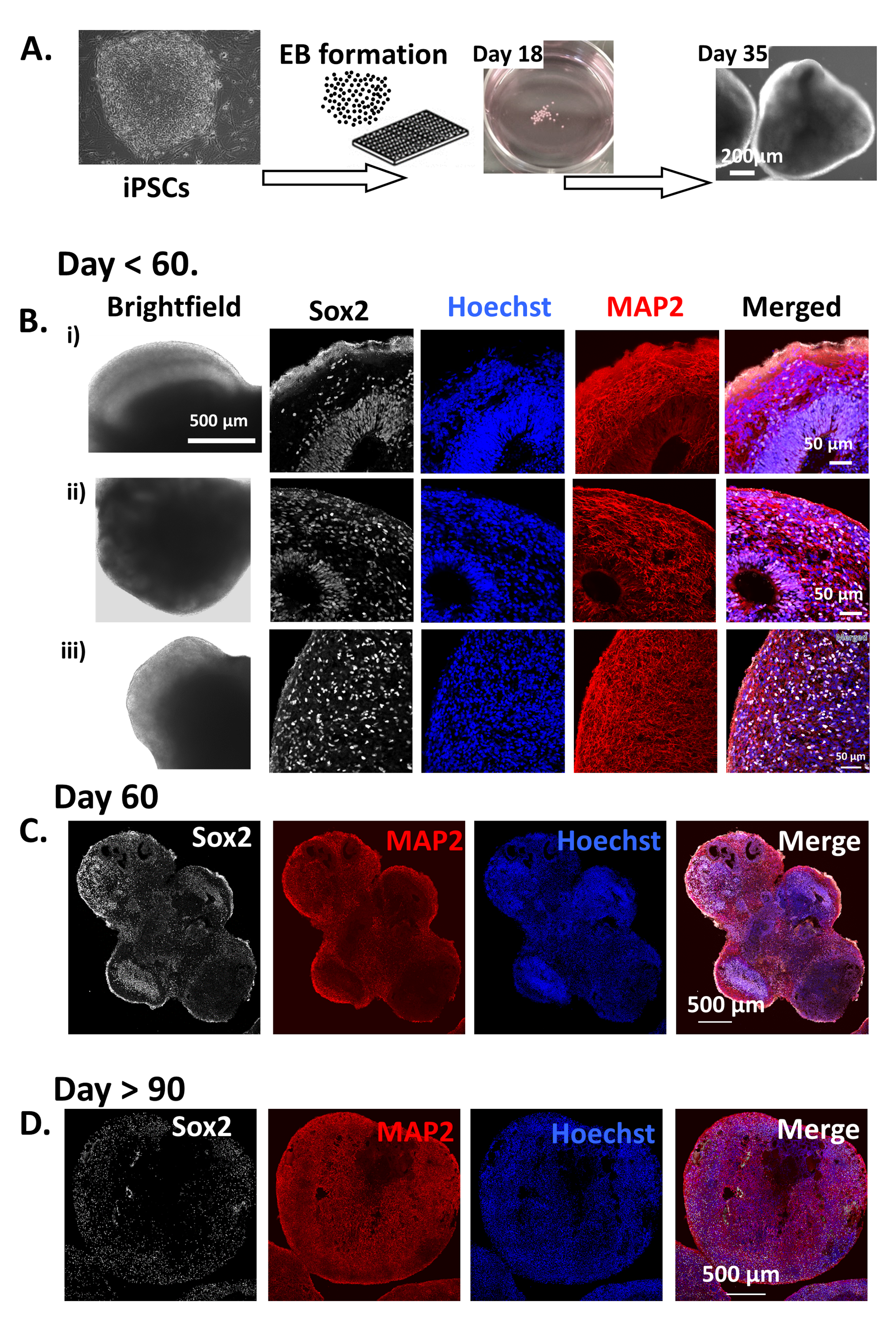

Supplement: S2 Fig — (A) The process of 3D culture protocol begins with iPSCs that are dissociated into a single-cell suspension and induced to form embryoid bodies (EBs) in a 96 well format (one EB per well). EBs were then differentiated over a period of weeks to months. (B) Images from organoids cultured for fewer than 60 days: phase contrast images (left) and sections immunostained using antibodies against markers of neural progenitor cells (SOX2, white) and neurons (MAP2, red), as well as the nuclear stain Hoechst (blue). In the images, one can see the (i) rolling morphology structure with organized translucent layers previously reported by the Sasai group [64], (ii) regions of the organoid with a rosette-like structure dense in neural progenitors, and (iii) unorganized translucent regions. (C and D) At 60 and 90 days in culture, organoids exhibit SOX2 (white) and MAP2 (red) immunoreactivity that is variable by region. (TIF) [file pone.0161969.s002.tif]

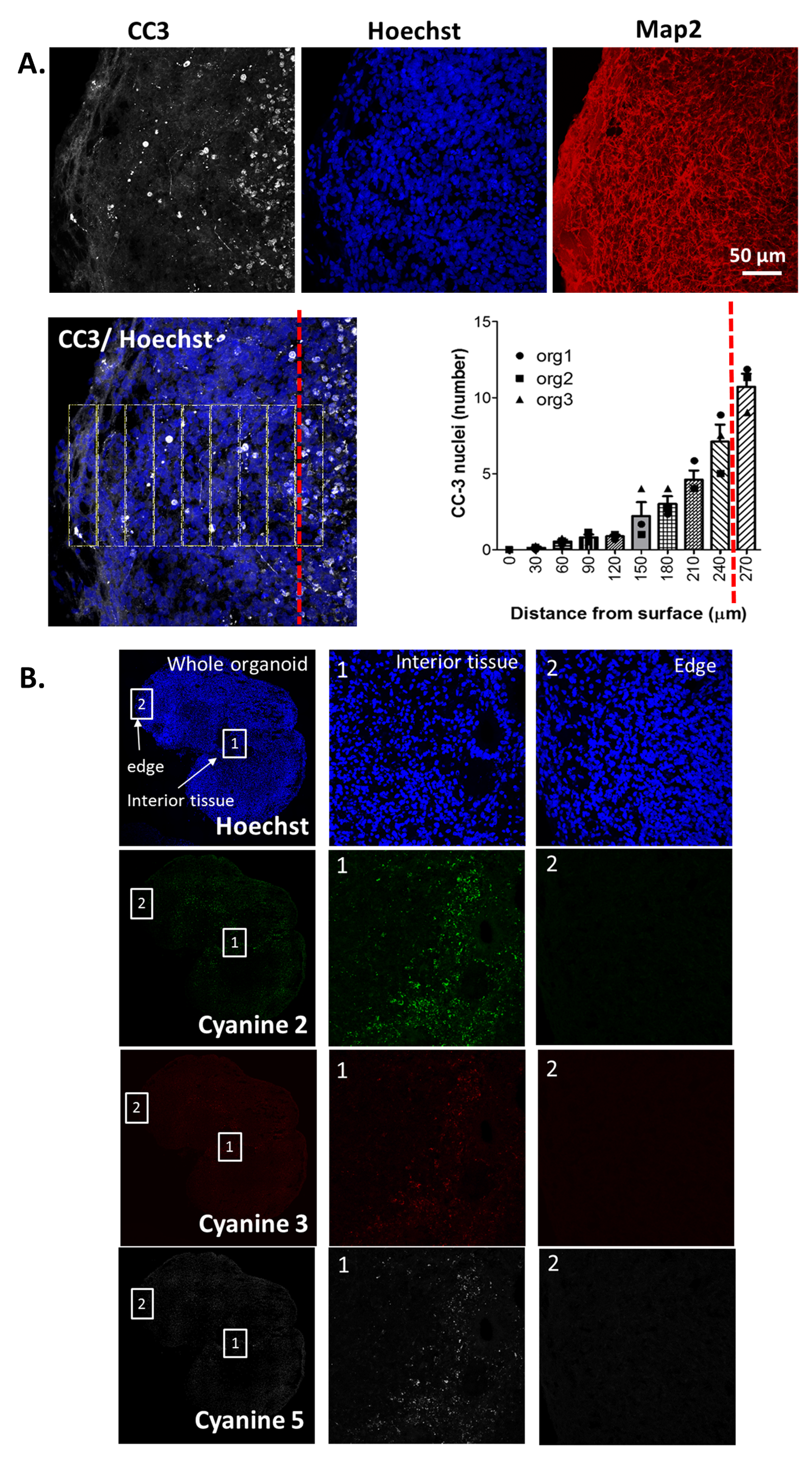

Supplement: S3 Fig — (A) A quantitative analysis of CC3 immunoreactivity leads us to limit our analyses to the peripheral 250 μm of MAP2-rich (red) and CC3-poor (white) region of the organoid. CC3- and Hoechst (nuclei)-positive cells were counted in bins of 30μm from the surface of the organoid in three replicates. (B) Organoid sections were blocked and incubated with secondary antibodies alone to determine the degree of non-specific labeling (left column = 90d whole organoid images). Regions of interest within the first 250 μm (right column) and deep (middle column) in the organoid tissue were analyzed for signal from secondary antibodies (anti-mouse-Cy2, green; anti-rabbit-Cy3, red; anti-chicken, white). (TIF) [file pone.0161969.s003.tif]

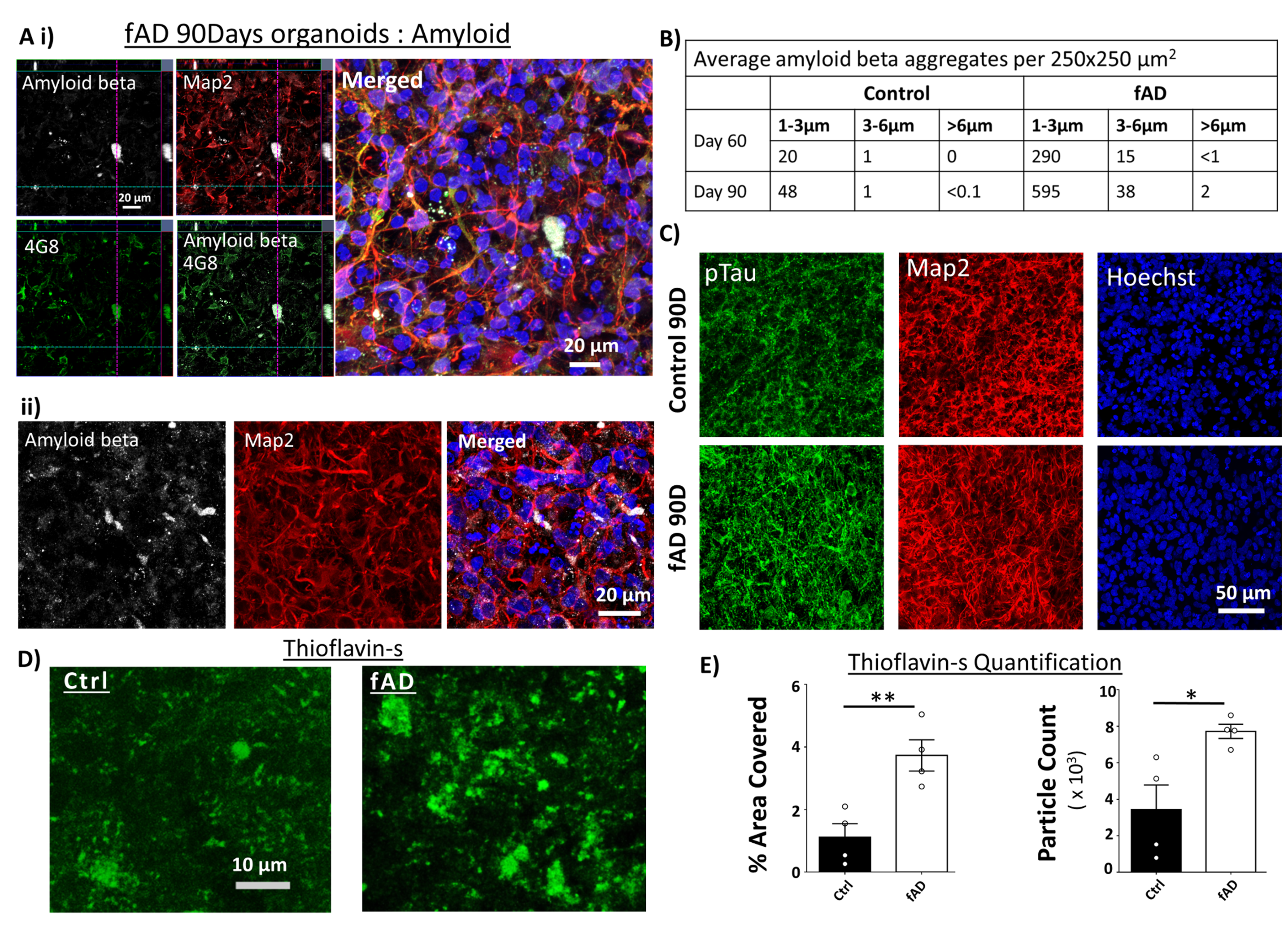

Supplement: S4 Fig — (A) Additional images from tissue sections from fAD (APPDp1-1) and control (Ctrl; CS-0020-01) organoids processed for immunoreactivity against amyloid β (Aβ) using two antibodies (D54D2: white, 4G8: green), as well as antibodies against the neuronal marker MAP2 (red) and stained with the nuclear dye Hoechst (blue). (B) Table of average particle number observed per 250 x 250 μm2 area in control and fAD organoids. (C) Tissue sections from fAD (APPDp1-1) and control (Ctrl; CS-0020-01) organoids were processed for immunoreactivity against phosphorylated Tau (pTau, green) at Thr181 and MAP2 (red) following 90 days of culture. Hoechst (blue) labels cell nuclei. (D) Representative images of fAD (APPDp1-1) and control (Ctrl; CS-0020-01) organoids section stained with Thioflavin-s dye for beta-pated sheet structure. (E) Quantification of fluorescent Thioflavin-S in tissue sections from fAD (APPDp1-1) and control (Ctrl; CS-0020-01) organoids after 90 days of culture (Each data point represent one organoid). Measurements consist of the percent of the visual field covered by Thioflavin-S signal (left) and the number of distinct Thioflavin S-positive particles (right). Unpaired two-tail t-test with equal variance, **p = 0.0074 (% Area Covered), *p = 0.022 (Particle Count). On charts: *p < 0.05, **p < 0.01. (TIF) [file pone.0161969.s004.tif]
