# Supplementary material for: Self-Organizing 3D Human Neural Tissue Derived from Induced Pluripotent Stem Cells Recapitulate Alzheimer’s Disease Phenotypes
Source: PLoS One. 2016 Sep 13;11(9):e0161969. doi: 10.1371/journal.pone.0161969 (PMC5021368; doi:10.1371/journal.pone.0161969)
Supplement: S1 Table — (PDF) [file pone.0161969.s007.pdf]

Table S1. Cell lines used in the current study

Cell line code	Source	Gender	Diagnosis	Genotype	Age at Biopsy	ApoE	Reports (PMID)	TRA-1-60	TRA-1-81
APP^{Dp}1-1	Goldstein, UCSD	Male	Familial Alzheimer's disease	*APP* duplication	51	3/3	22278060	✓	✓
APP^{Dp}2-3	Goldstein, UCSD	Female	Familial Alzheimer's disease	*APP* duplication	60	3/3	22278060	✓	✓
ND34732	Coriell	Female	Familial Alzheimer's disease	*PSEN1* M146I	33	2/3		✓	✓
AG06840	Coriell	Male	Familial Alzheimer's disease	*PSEN1* A264E	56	3/3		✓	✓
CS-0020-01	Cohen, McLean, HMS	Male	Healthy		23	3/3		✓	✓
AG09173	Yankner, HMS, Coriell	Female	Healthy		75	3/3		✓	✓
